# Vegetation Traced to Natural Causes

**Published:** 1849-02

**Authors:** 


VEGETATION TRACED TO NATURAL CAUSES.

An ingenious pamphlet on this subject, by Mr. Daniel Vaughan, has been placed upon our table. The author ascribes all vital phenomena in plants to electricity. Electrical currents from the roots to the leaves effect, in his opinion, all the transformations which take place in the vegetable kingdom. The currents are kept up by evaporation, which is continually going on from the leaves. In the decay, or transformation of a seed, all the conditions are supposed to exist necessary to create an electrical current. The decaying humus sets the currents in motion towards the young plant, and keeps up the action commenced by the germinating seed. The development of buds and leaves, the author also ascribes to galvanic, or electrical, action. In the winter, when the fermentation of humus is almost suspended, the sap remains at rest, or only moves very slowly.

The tendency of science is more and more to the reduction of phenomena to a few causes. Chemical philosophy is explaining many processes which were long held to be essentially vital. Whether this attempt of Mr. Vaughan to explain vegetation by these laws has been successful, it would be premature to decide. His pamphlet shows a decided talent for such investigations.

